# Immunometabolic programming of macrophages in asthma pathogenesis and therapy

**DOI:** 10.3389/fphys.2025.1736340

**Published:** 2026-01-12

**Authors:** Lisha Lu, Mengdi Shi, Wen Qin, Mingshu Yang, Xiaochang Wang, Youpeng Wang

**Affiliations:** 1 Faculty of Clinical Medical College, Heilongjiang University of Chinese Medicine, Harbin, Heilongjiang, China; 2 Department of Pediatrics, The Second Affiliated Hospital of Heilongjiang University of Chinese Medicine, Harbin, Heilongjiang, China

**Keywords:** asthma, glycolysis, immunometabolism, lipid metabolism, macrophage

## Abstract

Asthma is a heterogeneous chronic airway disease in which immune dysregulation and metabolic imbalance jointly shape inflammatory phenotypes and clinical outcomes. Growing evidence identifies pulmonary macrophages as central integrators of inflammatory cues and metabolic programs, linking acute exacerbations with long-term airway remodeling. Distinct tissue-resident and monocyte-derived macrophage subsets polarize along an M1–M2 spectrum and adopt glycolysis-dominated pro-inflammatory states or fatty acid oxidation-centered reparative states that differentially drive neutrophilic versus type 2-biased eosinophilic inflammation. Rewiring of arachidonic acid–derived eicosanoid synthesis and cholesterol handling further tailors macrophage effector functions and modulates responsiveness to glucocorticoids. Preclinical studies demonstrate that pharmacological manipulation of macrophage glucose and lipid metabolism can attenuate airway hyperresponsiveness and structural remodeling, highlighting immunometabolic circuits as promising therapeutic targets in asthma. This review summarizes current advances in macrophage ontogeny, polarization and metabolic reprogramming in the asthmatic lung. It also discusses how these insights may inform metabolism-focused, macrophage-directed interventions.

## 1 Introduction

Asthma is the world’s second most prevalent chronic respiratory disease and affects more than 300 million people (GBD Chronic Respiratory Disease Collaborators, 2020; Papi et al., 2018). Its hallmark is reversible airflow limitation that develops in both adults and children and results from a complex interplay between genetic predisposition and environmental exposure. Risk factors such as microbial contact, environmental pollution, tobacco smoke, obesity, and a positive family history contribute to the marked heterogeneity of asthma and give rise to multiple phenotypes with distinct clinical presentations and pathological features (Beasley et al., 2015). Wheeze, dyspnea, chest tightness, and cough arise from airway obstruction, airway hyperresponsiveness, and airway inflammation. Immune responses initiated by the synergy of genetic susceptibility and environmental triggers are central to disease pathogenesis and are accompanied by metabolic dysregulation and cellular dysfunction (Papi et al., 2018).

Airway inflammation is predominantly driven by T helper 2 (Th2)-dependent mechanisms that involve immunoglobulin E (IgE), IgE secreting B cells, mast cells, and eosinophil recruitment (Holgate, 2012). Beyond allergen specific Th2 mediated adaptive immunity, macrophages act as pivotal innate immune cells in both allergic and non allergic asthma (Chatila, 2016). They are involved in the development of a severe asthma phenotype characterized by T helper (Th) 1/Th17-skewed immune responses, neutrophilic airway inflammation, and poor responsiveness to glucocorticoid therapy. The pathological basis of this phenotype is closely associated with M1-polarized macrophages and metabolic reprogramming (Chen et al., 2025). Notably, macrophage metabolic reprogramming under asthmatic conditions is closely linked to acute exacerbations (Saradna et al., 2018). Experimental models show that asthmatic macrophages upregulate glycolytic enzymes and glycolytic flux, accumulate lactate, and increase pyroptosis markers, indicating that glycolytic reprogramming sustains their pro inflammatory activity (Chen et al., 2024). Lipid mediator biosynthesis in macrophages is equally plastic. For example, house dust mite (HDM) exposure can reprogram macrophage transcription through the formyl peptide receptor 2 (FPR2)/tumor necrosis factor (TNF)/2 HG/prostaglandin E_2_ (PGE_2_)–prostaglandin E_2_ (EP2) axis. This process leads to excessive secretion of TNF α, C-C motif chemokine ligand 17 (CCL17), leukotrienes, PGE_2_ and interleukin-6 (IL-6), and fosters a CCL17 high, M2 like phenotype that amplifies systemic allergic inflammation (Lechner et al., 2022). This review synthesizes current knowledge of macrophage subsets and lineages in asthma and highlights their metabolic reprogramming, with an emphasis on glucose and lipid metabolism. Finally, it discusses the therapeutic potential of targeting macrophage metabolic pathways as a strategy for metabolic intervention in asthma.

## 2 Pulmonary macrophage lineages and their phenotypic plasticity

Lung macrophages comprise two principal populations. During embryogenesis, fetal monocytes seed the neonatal lung and differentiate into tissue resident alveolar macrophages (TRAMs), which are self-maintained under homeostatic conditions (Satoh et al., 2013). When TRAMs are injured or depleted, circulating monocytes are recruited and mature into monocyte derived alveolar macrophages (MoAMs). Together, these two cell types rebuild the alveolar niche (Hashimoto et al., 2013). Experimental evidence shows that Mo AMs dominate during acute inflammation and pulmonary fibrosis and can persist long term in the lung (Misharin et al., 2017). In the interstitium, less-studied interstitial macrophages (IMs) can be further divided into IM1 (MHC class II^low^ CD206^high^), IM2 (MHC class II^+^ CD206^high^) and IM3 (MHC class II^high^ CD206^low^ CCR2^+^) subsets (Gibbings et al., 2017). Under steady-state conditions, TRAMs are characterized by high expression of CD11c, MARCO, CD169, and other markers, and are adapted to the lipid-rich, low–pathogen load environment of the alveolar space. They are primarily responsible for clearing surfactant and cellular debris, maintaining epithelial integrity, and supporting immune tolerance. Interstitial macrophages (IMs) can be subdivided, based on markers such as MHCII, Lyve-1, and CD36, into subsets that are more biased toward antigen presentation or toward repair and regulation. These populations are distributed in perivascular and peribronchial niches, where they contribute to stromal support and the production of IL-10, among other functions (Bain and MacDonald, 2022). During infection, inhalational injury, or severe inflammation, a proportion of tissue-resident AMs and IMs is lost or functionally compromised (Bain and MacDonald, 2022). Circulating classical monocytes are then continuously recruited into the lung and differentiate into monocyte-derived AMs or IMs. A fraction of these cells persists long term after resolution of inflammation and, under the influence of the lung tissue milieu, gradually acquires phenotypic and transcriptional features resembling those of resident macrophages, thereby replenishing or replacing the preexisting resident pool (Bain and MacDonald, 2022). Further study has shown that classical monocyte subsets with distinct developmental origins can seed the lung interstitium at steady state and after immune challenge, differentiating into phenotypically and functionally diverse IM subsets. This directly links monocyte developmental lineages to the heterogeneity of lung tissue-resident macrophages (Trzebanski et al., 2024). In allergic asthma, allergen exposure rapidly recruits monocytes that differentiate into Mo AMs to amplify acute inflammation, whereas TR AMs proliferate locally and restrain allergic responses. The two populations therefore cooperate to maintain airway immune balance (Draijer and Peters-Golden, 2017). Macrophages are highly plastic cells whose activities are shaped by extracellular cues, and their phenotypes form a continuum that extends from the pro inflammatory M1 pole to the anti inflammatory and reparative M2 pole.

### 2.1 Classical (M1) activated macrophages in asthma

**FIGURE 1:**
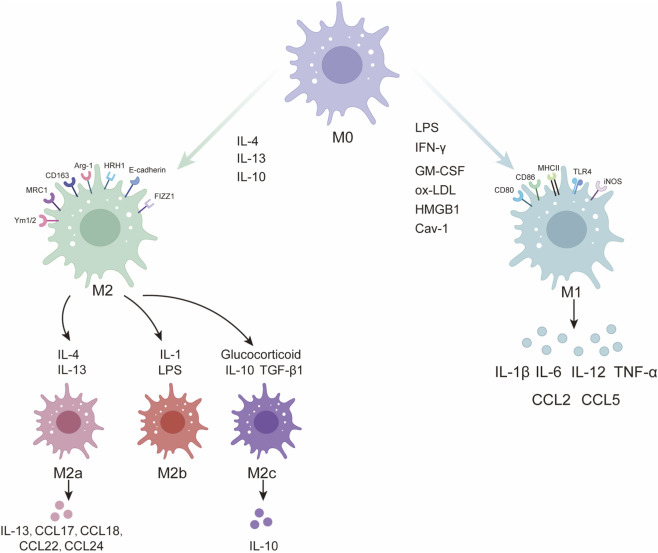
Polarization and Functions of Macrophages. LPS, IFN-γ, ox-LDL, HMGB1, Cav-1 and GM-CSF drive macrophages toward the M1 phenotype. M1 cells characteristically express CD80, CD86, MHC II, TLR4 and inducible iNOS and release large amounts of pro-inflammatory Th1 cytokines such as IL-6, IL-12, IL-1β and TNF-α together with chemokines including CCL2 and CCL5. Interleukin-4, interleukin-13 or interleukin-10 induce M2 polarization. Canonical M2 macrophages display high levels of the MRC1, CD163, Arg-1, HRH1, E-cadherin, Ym1/2 and FIZZ1. Based on the inducing stimulus, M2 macrophages subdivide into M2a, M2b and M2c subsets: M2a cells arise from IL-4 or IL-13, M2b cells from IL-1 receptor ligands or immune complexes combined with LPS, and M2c cells from IL-10, transforming growth factor-β1 or glucocorticoids. M2a cells secrete abundant IL-13 and chemokines CCL17, CCL18, CCL22 and CCL24, which activate Th2 cells and recruit eosinophils to the lung, whereas M2c cells release large quantities of the anti-inflammatory cytokine IL-10. LPS, lipopolysaccharide; IFN-γ, interferon-γ; ox-LDL, oxidized low-density lipoprotein; HMGB1, high-mobility group box 1; Cav-1, caveolin-1; GM-CSF, granulocyte-macrophage colony-stimulating factor; MHC II, major histocompatibility complex class II; iNOS, inducible nitric-oxide synthase; IL, interleukin; TNF-α, tumor necrosis factor-α; MRC1, mannose receptor C-type 1; Arg-1, arginase-1; HRH1, histamine H1 receptor; Ym1/2, chitinase-like proteins Ym1 and Ym2; FIZZ1, resistin-like molecule α; TGF-β1, transforming growth factor-β1; Th1, T helper type 1; Th2, T helper type 2.

Lipopolysaccharide (LPS), interferon γ (IFN γ), and granulocyte macrophage colony stimulating factor efficiently drive macrophages toward the M1 phenotype (Figure 1) (Saradna et al., 2018). More recent work identifies oxidized low density lipoprotein (Qi et al., 2021), high mobility group box 1 (He et al., 2021) and caveolin 1 (He et al., 2021) as additional M1 regulators. M1 macrophages characteristically express CD80, CD86, MHC class II, toll-like receptor 4 (TLR4), and inducible nitric oxide synthase (iNOS). They secrete abundant T helper 1 cytokines (IL 6, IL 12, IL 1β, TNF α) together with chemokines such as CCL2 and CCL5. Their primary roles include intracellular pathogen clearance and recruitment and activation of T and B lymphocytes. Allergic and non allergic asthma both feature increased macrophage numbers, but their polarization status differs. Robbe and colleagues compared an HDM allergic model with a farm dust extract non allergic model (Robbe et al., 2015). The non allergic model was dominated by M1 macrophages and displayed enhanced Th1 and Th17 responses, whereas the allergic model exhibited M2 polarization and a typical Th2 profile. These observations suggest that M1 macrophages are key effector cells in non allergic asthma, whereas M2 cells predominate in allergic disease. M1 polarization is also closely associated with severe asthma, particularly in patients who respond poorly to systemic glucocorticoids (Saradna et al., 2018).

Within the asthmatic airway microenvironment, M1-polarized macrophages exhibit robust phagocytic activity and a pronounced pro-inflammatory phenotype. However, in atopic patients the bacterial and viral burden detected in the airways is typically insufficient to be effectively controlled by their antimicrobial functions (Fricker and Gibson, 2017; Blasi and Johnston, 2007). Instead, mediators and signaling pathways associated with the M1 program correlate closely with disease activity and therapeutic responsiveness. Clinical and experimental data demonstrate markedly elevated macrophage-derived NO in exhaled breath condensate of patients with asthma (Khanduja et al., 2011; Naura et al., 2010). High NO levels induce oxidative DNA damage and, in allergen-sensitized murine models, augment mucus secretion, thereby exacerbating airway obstruction. Concurrently, M1 macrophages secrete IL-1α, IL-1β, IL-6, and TNF-α, which activate CD4^+^ T cells and upregulate IL-5. The latter is pivotal for eosinophil proliferation, migration, and degranulation, sustaining airway inflammation and hyper-responsiveness. IL-6 also promotes fibroblast proliferation, activates the MAPK cascade, and synergizes with TGF-β to drive fibrotic remodeling (Gallelli et al., 2008; Li et al., 2022). Potent pathogen-associated molecular patterns such as high-dose LPS elicit a type 1 inflammatory phenotype characterized by neutrophilic inflammation, airway hyper-reactivity, enhanced IL-12 expression, enrichment of CD11b^high^ F4/80^high^ (CD11c-variable) inflammatory macrophages, and upregulation of IL-27 (Kim et al., 2007; Li et al., 2010; Moreira and Hogaboam, 2011). In glucocorticoid-resistant asthma, IL-27 and IFN-γ are concomitantly elevated; together they activate a MyD88-dependent pathway that impedes nuclear translocation of the glucocorticoid receptor, thereby reducing steroid sensitivity. IL-27 further induces IFN -stimulated genes and a STAT1-dominated pro-inflammatory transcriptional program in monocytes/macrophages, amplifies Toll-like-receptor responsiveness, and suppresses IL-10 signaling, collectively expanding inflammatory cascades and reinforcing M1 polarization (Li et al., 2010; Khalil et al., 2023; Kalliolias and Ivashkiv, 2008). Cytokines traditionally linked to the Th2 axis can also potentiate M1 traits under defined conditions. IL-4, via MyD88 signaling, increases macrophage expression of IL-6, TNF-α, and IL-12, whereas IL-33, although boosting arginase-1, Ym-1, and mannose receptor in pre-polarized M2 cells, elevates the M1-associated chemokine CCL3 in unpolarized macrophages (Varin et al., 2010; Joshi et al., 2010). Overall, the central connection between M1 polarization and asthma resides in the combined burden of reactive oxygen/nitrogen species, pro-inflammatory cytokines, amplified innate-immune signaling, and negative modulation of glucocorticoid responses. These factors together perpetuate chronic airway inflammation, structural remodeling, and the emergence of treatment-refractory phenotypes.

### 2.2 Alternative (M2) activated macrophages in asthma

Unlike IFN-γ or LPS activated M1 macrophages, M2 polarization is induced by IL-4, IL-13 or IL-10. Depending on the stimulus, M2 macrophages are classified into M2a, M2b and M2c subsets (Luo et al., 2024). IL-4/IL-13 or fungal and helminth infections induce M2a cells; IL-1 receptor ligands or immune complexes combined with LPS generate M2b cells; IL-10, TGF-β1 or glucocorticoids produce M2c cells. Typical M2 macrophages express high levels of the mannose receptor (MRC1), CD163 and arginase-1 (Arg-1) and low levels of iNOS, MHC II and CD86 (Luo et al., 2024; Bogie et al., 2014). Histamine H1 receptor (HRH1) and E-cadherin are highly expressed on cultured M2 cells and on M2 macrophages from broncho-alveolar lavage fluid (BALF) of asthma patients, serving as auxiliary markers (Girodet et al., 2016); Ym1/2 and FIZZ1 are also commonly used markers (Raes et al., 2002; Kang et al., 2022). Functionally, M2a cells secrete abundant IL-13 and chemokines such as CCL17, CCL18, CCL22 and CCL24, which activate Th2 cells and recruit eosinophils to the lung (Deng et al., 2023; Brollo et al., 2024). M2c cells release high levels of the anti-inflammatory cytokine IL-10 and exhibit low nuclear factor κ-B (NF-κB) activity as well as reduced CD40, CD86 and HLA-DR expression (Jiang and Zhu, 2016). Because IL-4 and IL-13 are central inducers of M2 polarization, M2 macrophages are regarded as principal effector cells in allergic asthma. Recent work shows that IL-33 modulates M2 polarization through its receptor ST2, which also binds IL-4, IL-5, IL-13, CCL17, CCL18 and CCL24 (Faas et al., 2021; Sheng et al., 2025). Eosinophils, group 2 innate lymphoid cells, CD4^+^ CD25^+^ regulatory T cells and mesenchymal stem cells have also been reported to drive M2 polarization (Tiemessen et al., 2007; You et al., 2020; Zhang et al., 2015; Luque-Campos et al., 2021). Girodet and colleagues demonstrated that BALF from asthma patients contains markedly more M2 macrophages than that from healthy controls, with MRC1 and MHC II expression increased by more than threefold (Lai et al., 2023). These observations indicate that targeting M2 polarization and function is a promising therapeutic strategy that may act synergistically with current treatments. The binary M1/M2 model largely derives from studies in which macrophages are exposed to single, defined stimuli *in vitro* and is therefore a useful simplification for dissecting signaling pathways and effector functions (Viola et al., 2019). However, under physiological conditions macrophages are simultaneously exposed to numerous cytokines, growth factors, lipids and microbial or danger signals within the tissue microenvironment, and as a result they only rarely display purely prototypical M1-or M2-like profiles (Viola et al., 2019). Instead, most tissue-resident and recruited macrophages occupy a spectrum of intermediate activation states with overlapping transcriptional and functional features, and their phenotype at any given time reflects the integrated sum of local cues rather than a fixed subset identity (Li et al., 2019). Culture conditions can also reshape macrophage characteristics compared with their *in-situ* counterparts, which further limits direct extrapolation from *in vitro* polarization systems (Brykczynska et al., 2020). To describe macrophage states more accurately, researchers have proposed alternative classification schemes based on inducing stimuli, tissue-specific functions or resemblance to T helper subsets, though each has its own limitations (Brykczynska et al., 2020). It is noteworthy that both polarized M1 and M2 macrophages serve as efficient antigen-presenting cells. Through distinct cytokine profiles and costimulatory molecules such as CD86 and MHC II, they guide the differentiation of Th1, Th2, Th17 and regulatory T cells, thereby orchestrating both non-allergic and allergic airway inflammation (Venosa et al., 2016).

In the methyl-mustard injury model, macrophage polarization is initially skewed toward M1 during days 1–3 post-injury, whereas M2 cells peak by day 28 in parallel with progressive pulmonary fibrosis (Pauleau et al., 2004). At this stage, M2 macrophages exhibit marked upregulation of arginase-1, Ym1/2, Fizz1/RELM-α, and the mannose receptor CD206, collectively driving allergic inflammation toward structural damage. Arginase-1, induced by IL-4/IL-13, can increase its transcript levels by more than three orders of magnitude (Pauleau et al., 2004); by competing for L-arginine, it limits iNOS-derived nitric oxide (NO), thereby attenuating bronchodilation, whereas its product ornithine fuels polyamine synthesis through the ornithine decarboxylase (ODC) pathway to enhance airway hyper-responsiveness and supplies proline for collagen deposition that accelerates remodeling (Rupani and Kent, 2022; Sen et al., 2023; North et al., 2013). Ym1/2 binds heparan sulfate or GlcN oligomers, functioning both in pathogen recognition and eosinophil chemotaxis, thus amplifying inflammation and participating in matrix reorganization (Shuhui et al., 2009; Webb et al., 2001). Fizz1, sharply increased in a STAT6-dependent manner, directly engages fibroblasts to induce type I collagen and α-SMA expression, promoting peribronchial fibrosis and epithelial thickening (Zhang et al., 2009; Doherty et al., 2012). CD206 is upregulated by IL-4/IL-13 and potentiated by estrogen; via miR-511-3p it stabilizes the alternatively activated phenotype, whereas loss of either CD206 or miR-511-3p shifts macrophages toward M1 and worsens inflammation, and elevated CD206^+^M2 counts correlate with poor steroid responsiveness (Draijer et al., 2017; Zhou et al., 2018).

Accumulating evidence indicates that the composition of macrophage subsets and their M1/M2 polarization status not only shape the immunophenotype of asthmatic inflammation, but also profoundly influence the optimal therapeutic strategies at different disease stages (Draijer and Peters-Golden, 2017). In allergic asthma, allergen exposure rapidly mobilizes circulating monocytes and promotes their polarization toward proinflammatory-like macrophages, whereas multiple studies suggest that TRAMs often exhibit more anti-inflammatory and tissue-protective phenotypes (Misharin et al., 2017; Draijer and Peters-Golden, 2017). Within the same lung, several interstitial macrophage subsets also coexist, and dynamic changes in these lineages and their relative proportions are closely associated with the intensity of inflammation and the extent of long-term airway and pulmonary parenchymal remodeling (Misharin et al., 2017; Gibbings et al., 2017). In early or mild-to-moderate asthma characterized by a typical Th2-high endotype with predominant eosinophilic inflammation but minimal structural remodeling, experimental data suggest that, while preserving essential antimicrobial functions, skewing TRAMs and a fraction of recruited macrophages toward a regulatory M2-like phenotype characterized by IL-10 production and moderate levels of TGF-β may help prevent excessive amplification of M1/Th1 and M2a/Th2 responses (Misharin et al., 2017; Gibbings et al., 2017).

In a cockroach allergen–induced murine model, Mrc1 deficiency reduces miR-511-3p levels in alveolar macrophages and drives their shift toward an M1 phenotype, accompanied by increased Th2- and Th17-associated cytokines and exacerbated airway inflammation (Zhou et al., 2018); conversely, adeno-associated virus–mediated delivery of miR-511-3p enhances the expression of M2 markers such as Arg1, attenuates inflammatory cell infiltration, and alleviates airway hyperresponsiveness. These findings support a protective role for moderate enhancement of the MRC1–miR-511-3p–related regulatory M2 pathway in early allergic asthma (Zhou et al., 2018; Do et al., 2019). Mechanistic studies further demonstrate that miR-511-3p downregulates multiple proinflammatory genes by inhibiting the CCL2–CCR2–RhoA signaling axis and dampening CCL2-induced M1 polarization, thereby providing a druggable molecular target to selectively redirect macrophages toward a more anti-inflammatory, resolution-promoting M2-like phenotype (Do et al., 2019). By contrast, when disease progresses to a chronic stage with overt airway remodeling or pulmonary fibrosis, long-lived monocyte-derived macrophages within the lung exhibit a transcriptional profile enriched for M2/profibrotic gene signatures, and their accumulation correlates positively with collagen deposition and the severity of lung fibrosis, suggesting that in this phase it may be necessary to inhibit or reprogram these pathological M2-like macrophages to mitigate structural damage (Misharin et al., 2017). Consistent with this notion, in an ovalbumin (OVA)-induced murine model of chronic allergic airway inflammation, low–dose-rate ionizing radiation selectively reduces the proportion of CD206-positive M2-like macrophages in bronchoalveolar lavage fluid and lung tissue, accompanied by decreased IL-4 and IL-13 levels and amelioration of inflammatory responses. These data suggest that, at stages dominated by M2 responses and structural remodeling, moderate attenuation of M2-associated signaling may help control persistent Th2-driven inflammation and mucus hypersecretion (Jo et al., 2022). Another study using a chronic OVA exposure model shows that systemic administration of extracellular vesicles derived from hypoxia-preconditioned human umbilical cord mesenchymal stem cells significantly reduces inflammatory cell counts and IL-4 and IL-13 levels in bronchoalveolar lavage fluid, and suppresses TGF-β1/Smad2/3 signaling as well as α-SMA and type I collagen expression. Mechanistically, miR-146a-5p, which is enriched in these vesicles, is upregulated in lung tissue and fibroblasts and modulates relevant pathways, thereby broadly attenuating the M2-associated profibrotic factor network and highlighting the translational potential of fine-tuning M2-related signaling via exogenous vesicles during the remodeling phase (Dong et al., 2021).

Beyond the classical M1/M2 dichotomy, an IL-9–producing macrophage subset, termed M(IL-33+IL-2), has been identified in OVA-induced allergic airway inflammation models. This subset, driven by IL-33 alone or in combination with IL-2, can be induced both in murine lungs and in human monocyte-derived macrophages, and markedly amplifies eosinophilic inflammation and mucus secretion, suggesting that targeting the IL-33–IL-2–IL-9 axis to suppress this specific pathogenic macrophage subset represents another potential avenue for precision therapy (Guo et al., 2025). Taken together, these observations indicate that in patients with asthma of different etiologies and endotypes, therapeutic strategies targeting macrophage polarization will likely need to be differentiated and tailored to disease stage.

## 3 Cell intrinsic metabolic reprogramming in airway macrophages

Accumulating evidence indicates that, in the asthmatic airway, macrophages rapidly rewire both glycolytic and lipid pathways. This metabolic shift amplifies their pro-inflammatory capacity, contributes to airway remodeling, and compromises steroid responsiveness (Albers et al., 2025; Maier et al., 2023). Clinical and preclinical studies show a positive correlation between an enhanced glycolysis–lactate axis and disease severity. Pharmacological inhibition of this pathway alleviates airway injury (Chen et al., 2024). Allergen-driven upregulation of macrophage glycolysis has also emerged as a pivotal driver of type 2 immunity (Albers et al., 2025). On the lipid side, HDM exposure reroutes arachidonic acid metabolism toward pro-inflammatory leukotrienes and 12/15-LOX products, further intensifying airway inflammation (Henkel et al., 2019). Conversely, boosting cholesterol efflux via ATP-binding cassette transporter A1 (ABCA1) dampens neutrophilic inflammation, underscoring the modulatory role of lipid homeostasis. Therefore, we reviewed recent studies on macrophage metabolic reprogramming in asthma and summarized the key findings in Table 1.

**TABLE 1:** Metabolic and lipid re-programming of macrophages in asthma.

Metabolic pathway	Experimental model	Macrophage subtype/cell line	Key metabolic changes	Pharmacological effects	References
Glycolysis	OVA-induced allergic asthma mice	F4/80^+^ lung macrophages	HK2, PKM2, LDHA, Lactate↑	Dexamethasone suppresses the HIF-1α–glycolysis–lactate axis, lessening airway inflammation	Chen et al. (2024)
	HDM-induced asthma mice	Alveolar macrophages	Baseline ECAR, Glycolysis↑	Macrophage targeted glycolysis blockade normalises airway hyper-responsiveness	Albers et al. (2025)
	Human allergen-challenge	Alveolar macrophages	Glycolysis gene↑	-	Alladina et al. (2023)
	OVA-challenged asthma mice	Lung macrophages	TLR4/MyD88/NF-κB-driven glycolysis boost; Lactate ↑	TLR4/MYD88/NF-κB–driven macrophage glycolysis promotes allergic asthma development	Ji et al. (2024)
	LPS-induced NR8383 cells	NR8383 alveolar macrophages	ECAR, Lactate, PKM2, LDHA ↑; M1 bias	Targeting PKM2 attenuated LPS-induced glycolysis and M1 polarization in NR8383 cells, thereby alleviating lung injury	Xiang et al. (2024)
	Formaldehyde + OVA/LPS asthma mice	Monocyte-derived macrophages	LDHA, Lactate ↑	Glycolysis inhibitors dampen FA/LPS-induced TNF-α, IL-6, IL-1β production and lessen allergic asthma exacerbation	Xuan et al. (2024)
Lipid	AhR-knockout mice	Alveolar macrophages	PTGS1/COX-1, ALOX5 ↓; PGE_2_/LTC_4_ imbalance	AhR deficiency–induced steroid dysregulation exacerbates HDM–triggered allergic airway inflammation	Henkel et al. (2019)
	HDM-allergic mice and asthma patients	Alveolar macrophages	2-HG and PGE_2_/EP2 signaling, CCL17 secretion ↑	HDM-induced *in vitro* macrophage training is driven by the FPR2–TNF–2-HG–PGE2/EP2 axis, leading to an M2-like phenotype with high CCL17 production	Maier et al. (2023)
	OVA-induced asthma mice	Alveolar macrophages	Cholesterol efflux via ABCA1 ↑; G-CSF ↓	ABCA1 expression lowers neutrophilic airway inflammation	Lechner et al. (2022)
	CLP-deficient mice challenged with HDM	Alveolar macrophages	PGD_2_, CRTH2 pathway ↑	CLP restitution reduces PGD_2_ and airway inflammation	Dai et al. (2014)
	Macrophages exposed to 12,13-diHOME	THP-1 human monocytes	IL-1*β* ^high^CD206^low^ macrophages ↑	Exposure to 12,13-diHOME remodels macrophage chromatin, restricts ISRE accessibility, dampens LPS-induced interferon-responsive genes, and intensifies allergen-driven inflammation	Lin et al. (2024)

### 3.1 Aerobic glycolysis and lactate signaling in macrophage dependent airway inflammation

**FIGURE 2:**
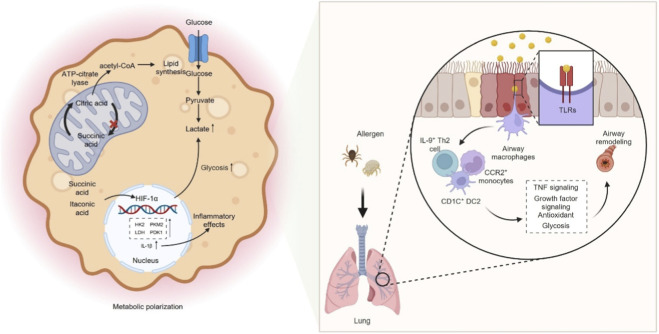
Glycolysis–dependent macrophage polarization and airway remodeling. M1-polarized macrophages rely on high-rate aerobic glycolysis, whereas alternatively activated (M2-polarized) macrophages maintain an intact TCA cycle and fatty acid–driven OXPHOS. In M1 macrophages, the TCA cycle is functionally interrupted at the citrate and succinate nodes: citrate is exported and cleaved by ATP-citrate lyase to provide acetyl-CoA for lipid mediator synthesis and histone acetylation, whereas accumulation of succinate, together with the mitochondrial metabolite itaconate, stabilizes HIF-1α, enhances ROS production, and drives expression of glycolytic enzymes and IL-1β. Mechanistically, succinate accumulation inhibits prolyl hydroxylases and stabilizes HIF-1α, which in turn upregulates key glycolytic enzymes such as hexokinase-2, pyruvate kinase M2, and lactate dehydrogenase, and induces pyruvate dehydrogenase kinase 1, thereby enhancing lactate production, limiting pyruvate entry into the TCA cycle, and providing ATP and NADPH to sustain inflammatory effector functions. Allergens can promote M1-like activation in a TLR-dependent manner, characterized by coordinated upregulation of both glycolysis and OXPHOS. IL-9^+^ Th2 cells, CD1C^+^ DC2 cells, and CCR2^+^ monocyte-derived cells accumulate in asthmatic airways, amplify TNF signaling, suppress antioxidant and growth factor cascades, and enforce glycolytic reprogramming, thereby linking type 2 inflammation to airway remodeling. M1, classically activated macrophage; M2, alternatively activated macrophage; TCA, tricarboxylic acid; OXPHOS, oxidative phosphorylation; ATP, adenosine triphosphate; ROS, reactive oxygen species; IL-1β, interleukin-1 beta; HIF-1α, hypoxia-inducible factor-1 alpha; NADPH, nicotinamide adenine dinucleotide phosphate (reduced form); TLRs, toll-like receptors; IL-9, interleukin-9; Th2, type 2 helper T cell; CD1C, CD1c molecule; DC2, type 2 conventional dendritic cell; CCR2, CC chemokine receptor 2; TNF, tumor necrosis factor.

Macrophages act as frontline sentinels of their microenvironment and therefore must continually adjust their metabolic wiring to support activation and effector functions. In broad terms, pro-inflammatory, classically activated (M1-biased) macrophages rely on high-rate aerobic glycolysis, whereas alternatively activated (M2-biased) cells maintain an intact tricarboxylic acid (TCA) cycle and fatty acid–supported oxidative phosphorylation (OXPHOS). Early work showed that inflammatory stimuli divert arginine through inducible NO synthase to generate NO, while M2 macrophages use arginase-1 to channel arginine into ornithine, polyamines and proline for tissue repair (Modolell et al., 1995; Huang et al., 2014). Subsequent metabolomic studies refined this model by demonstrating that in M1 macrophages the TCA cycle is functionally interrupted at citrate and succinate: citrate is exported and cleaved by ATP-citrate lyase to provide acetyl-CoA for lipid mediators and histone acetylation, whereas accumulation of succinate, together with the mitochondrial metabolite itaconate, stabilized hypoxia-inducible factor-1α (HIF-1α), enhances reactive oxygen species (ROS) production and drives expression of glycolytic enzymes and IL-1β (Michaeloudes et al., 2020; Goretzki et al., 2023; Infantino et al., 2011; Infantino et al., 2014; Williams and O'Neill, 2018; Lauterbach et al., 2019; Infantino et al., 2013; Lampropoulou et al., 2016). This pro-inflammatory metabolic shift resembles the Warburg effect described in tumors and is schematically summarised in Figure 2 (Michaeloudes et al., 2020; Goretzki et al., 2023). By contrast, M2-polarized macrophages preserve oxidative metabolism and fatty acid oxidation to sustain anti-inflammatory and pro-repair programs, underscoring that macrophage polarization is tightly coupled to distinct metabolic states rather than changes in single pathways (Ren et al., 2019; Rich and Maréchal, 2010; Scialò et al., 2017). Mechanistically, succinate accumulation inhibits prolyl hydroxylases and stabilized HIF-1α, which in turn upregulates key glycolytic enzymes such as hexokinase-2, pyruvate kinase M2 and lactate dehydrogenase, induces pyruvate dehydrogenase kinase 1 and diverts glucose-6-phosphate into the oxidative branch of the pentose phosphate pathway, thereby enhancing lactate production, limiting pyruvate entry into the TCA cycle and providing both ATP and nicotinamide adenine dinucleotide phosphate (NADPH) to sustain inflammatory effector functions (Tannahill et al., 2013; O'Neill et al., 2016; Nagy and Haschemi, 2015; Haschemi et al., 2012; Kelly and O'Neill, 2015; Papandreou et al., 2006; Caslin et al., 2020).

Clinical and experimental evidence links this glycolytic shift to asthma. Elevated lactate levels are detected in the bronchial epithelium and correlate with immune activation during disease progression (Ostroukhova et al., 2012). In OVA-driven asthma, both F4/80^+^ lung macrophages and OVA stimulated THP 1 cells display increased expression of glycolytic enzymes, enhanced glycolytic flux, higher lactate, and raised pyroptosis markers, implicating glycolytic reprogramming in their pro inflammatory activity (Chen et al., 2024). Dexamethasone attenuates airway inflammation and tissue injury by suppressing the HIF 1α–glycolysis–lactate axis and subsequent protein lactylation, thereby lowering glycolytic rate, lactate output, and pyroptosis (Chen et al., 2024). During asthma development, airway macrophages (AMs) rapidly engage the glycolytic program and directly modulate type 2 immunity. Under basal conditions their low glycolytic tone restrains Th2 responses. In contrast, exposure to allergens such as HDM upregulates glycolytic enzymes and lactate production via TLR2, fueling inflammation and metabolic adaptation (Albers et al., 2025). Selective blockade of macrophage glycolysis mitigates HDM induced airway hyperresponsiveness and leukocyte infiltration, and metabolic remodeling of the airway is more pronounced in patients with allergic asthma (Albers et al., 2025). Consistent with this, Yurakova et al. demonstrated that HDM and its key components imprint murine myeloid cells with a distinct immunometabolic phenotype characterized by concurrent increases in glycolytic and respiratory capacity in a TLR4-dependent manner (Yurakova et al., 2022), indicating that allergen-driven M1-like activation can involve coordinated upregulation of both glycolysis and OXPHOS rather than a simple shift away from mitochondrial respiration. Single cell RNA sequencing reveals that, post allergen challenge, AMs from asthmatic patients upregulate glycolytic genes and activate matrix degradation and mucin metaplasia pathways. In contrast, non asthmatic controls preferentially mount reparative and antioxidant programs. IL 9^+^ Th2 cells, CD1C^+^ DC2 cells and CCR2^+^ monocyte derived cells accumulate in asthmatic airways. Through a Th2–macrophage–basal cell network they amplify TNF signaling, suppress antioxidant and growth factor cascades and enforce glycolytic reprogramming, thereby linking type 2 inflammation to airway remodeling (Alladina et al., 2023). In an allergic asthma model, calprotectin S100A8/A9 drives M1 polarization via the TLR4/MyD88/NF κB axis and markedly augments glycolysis, as indicated by raised lactate and glycolytic enzymes. Blocking S100A8/A9 or administering the glycolytic inhibitor 3 bromopyruvate reduces pro inflammatory skewing and glycolytic flux, improving airway inflammation and lung damage (Ji et al., 2024). Likewise, LPS stimulated NR8383 alveolar macrophages adopt an M1 profile with elevated extracellular acidification rate, lactate, PKM2 and lactate dehydrogenase A (LDHA). Ephedrine or a PKM2 inhibitor dampens this glycolytic surge and M1 commitment, underscoring PKM2 dependent glycolysis in asthma related injury (Xiang et al., 2024). In asthma models and patient-derived monocyte-derived macrophages facing concurrent respiratory infection, exposure to fatty acids intensifies LDHA expression and lactate production, heightening glycolysis and driving excessive TNF-α, IL-6, IL-1β and NO release; the glycolytic antagonist 2-deoxyglucose (2-DG) reverses these pro-inflammatory effects (Xuan et al., 2024). Altogether, glycolytic reprogramming in macrophages underlies the initiation, exacerbation and infection-complicated phases of asthma, providing both the energetic supply and signaling framework for inflammation and offering therapeutic nodes such as glycolytic blockade or inhibition of HIF-1α and PKM2.

### 3.2 Lipidomic remodeling: synthetic and oxidative networks governing macrophage function

#### 3.2.1 Lipid-synthesis pathways in macrophages

Lipid metabolism is organized around two major routes: *de novo* fatty acid synthesis and cholesterol biosynthesis. Both lipid classes are built from acetyl-coenzyme A, a two-carbon unit primarily generated from glycolysis-derived carbon skeletons (Sharpe and Brown, 2013; Floris et al., 2020). Anabolic control is exerted by the sterol regulatory element-binding protein (SREBP) signaling axis, which transcriptionally upregulates pivotal enzymes such as fatty acid synthase, acetyl-CoA carboxylase 1, and 3-hydroxy-3-methylglutaryl-CoA reductase (Holthuis and Menon, 2014). SREBP-1 predominantly drives fatty acid formation, whereas SREBP-2 preferentially governs cholesterol production, and liver X receptors (LXRs) further modulate SREBP activity and phospholipid composition to maintain balanced pools of fatty acids and sterols (Horton et al., 2002; Britt et al., 2023; Wong et al., 2006; Ishibashi et al., 2013). Fatty acids themselves are fundamental constituents of membrane phospholipids (Harayama and Riezman, 2018); when mobilized, they give rise to arachidonic acid (AA), a poly-unsaturated fatty acid whose oxidative catabolism produces leukotrienes and prostaglandins biosynthesis (Wang et al., 2021). AA is a major lipid metabolite in asthmatic lungs and seeds multiple downstream effectors: cytosolic phospholipase A_2_ liberates AA from phospholipids, after which the 5-lipoxygenase (5-LOX) machinery converts it into leukotrienes, while cyclo-oxygenase isoenzymes oxidise AA to prostaglandins (Rådmark et al., 2015; Rakonjac et al., 2006; Bergstroem et al., 1964; Zhang et al., 2023). Cholesterol in turn, can be processed further into bile acids, vitamin D, and a variety of steroid hormones (Králová et al., 2018).

#### 3.2.2 Pro-inflammatory lipid biogenesis as a determinant of macrophage effector programming

**FIGURE 3:**
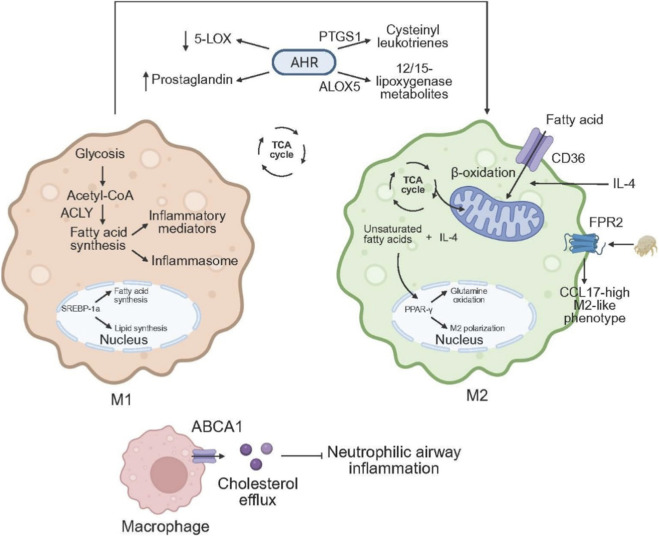
Lipid metabolic reprogramming linking macrophage polarization to asthma. In classically activated, M1-polarized macrophages, stimulation increases glycolytic flux and channels acetyl-CoA, via ATP-citrate lyase, into the fatty acid biosynthetic pathway. SREBP-1a is highly expressed and upregulates lipogenic genes such as FAS. This enhanced lipogenesis provides substrates for arachidonic acid–derived inflammatory mediators and amplifies inflammasome activity. In contrast, alternatively activated M2 macrophages maintain an intact TCA cycle and robust mitochondrial oxidative phosphorylation, deriving most of their energy from fatty acid uptake and subsequent β-oxidation. Fatty acids enter the cell through CD36, while IL-4 promotes intracellular triacylglycerol lipolysis to supply substrates for β-oxidation; unsaturated fatty acids cooperate with IL-4 to activate PPAR-γ, which further stabilizes M2 polarization. Brief exposure to HDM suppresses the 5-LOX pathway and activates COX-dependent prostaglandin production, whereas under sustained type 2 immune conditions lipid metabolism is skewed toward the generation of cysteinyl leukotrienes and 12-/15-lipoxygenase metabolites. AHR signaling fine-tunes this balance by transcriptionally regulating PTGS1 and ALOX5; loss of AHR reduces PGE_2_ and LTC_4_ levels and exacerbates airway inflammation. HDM can also reprogram macrophages via the FPR2–TNF–2-HG–PGE_2_–EP2 axis, increasing secretion of TNF, CCL17, leukotrienes, PGE_2_, and IL-6, and establishing a CCL17-high M2-like phenotype that amplifies systemic allergic responses. Overexpression of ABCA1 enhances cholesterol efflux from macrophages, reduces G-CSF production and neutrophil recruitment, and thereby attenuates neutrophilic airway inflammation.

*De novo* lipid synthesis is indispensable for plasma-membrane remodelling and for supplying precursors of pro-inflammatory mediators in classically activated (M1-biased) macrophages. Upon activation, these cells intensify glycolytic flux, generating ATP and citrate-derived acetyl-CoA that feeds the fatty acid biosynthetic machinery, as summarised in Figure 3; consistent with this demand, ATP-citrate lyase expression rises after stimulation, and its inhibition diminishes NO and reactive oxygen species production (Infantino et al., 2013; Dennis et al., 2010). Of the three SREBP isoforms, SREBP-1a is highly expressed in macrophages and accelerates their inflammatory program, since LPS upregulates SREBP-1a and Srebp1a-deficient mice exhibit impaired innate defences (Dinasarapu et al., 2013; Im et al., 2011). FAS is another pivotal node: myeloid-specific Fas deletion blocks macrophage infiltration into adipose tissue, dampens inflammation and shields mice from diet-induced insulin resistance (Wei et al., 2016). In combination, these data indicate that increased *de novo* fatty acid synthesis not only supplies substrates for eicosanoid production but also supports inflammasome activation and IL-1 family cytokine release, particularly in the presence of saturated fatty acids such as palmitate and appropriate priming signals (Batista-Gonzalez et al., 2019; Gianfrancesco et al., 2019; Korbecki and Bajdak-Rusinek, 2019; Wen et al., 2011). Consequently, in M1 macrophages lipid anabolism not only yields substrates for eicosanoid production but also amplifies inflammasome activity.

Alternatively activated (M2) macrophages, by contrast, preserve an intact tricarboxylic acid cycle and robust mitochondrial oxidative phosphorylation to support long-term energetic needs. Their catabolic energy arises from fatty acid uptake followed by β-oxidation (Odegaard and Chawla, 2011). Fatty acids enter via hydrolysis of circulating lipoproteins and CD36-mediated transport (Evans et al., 1993); (Huang et al., 2014), and interleukin-4 (IL-4) promotes intracellular triacylglycerol lipolysis to feed this pathway (Huang et al., 2014). Pharmacological inhibition of β-oxidation with the carnitine-palmitoyl transferase-1 (CPT1) blocker etomoxir suppresses IL-4-driven M2 programming, whereas enforced CPT1 activity helps maintain an anti-inflammatory phenotype even in the presence of palmitate (Mala et al., 2015). These oxidative programs depend on peroxisome proliferator-activated receptor-γ (PPAR-γ) and its co-activator PGC-1β, which coordinate fatty acid oxidation and support expression of hallmark M2 genes; unsaturated fatty acids cooperate with IL-4 to activate PPAR-γ, and PPAR-γ further sustains M2 polarization by enhancing glutamine oxidation (Banno et al., 2018; Vats et al., 2006; Odegaard et al., 2008; Nelson et al., 2018).

Macrophage lipid-mediator synthesis is highly plastic. Brief exposure to house-dust mite suppresses the 5-lipoxygenase pathway while activating cyclo-oxygenase-dependent prostaglandin production; persistent type-two conditions, such as allergic airway inflammation or helminth infection, shift toward cysteinyl-leukotrienes and 12/15-lipoxygenase metabolites (Henkel et al., 2019). Aryl-hydrocarbon-receptor signaling fine-tunes this balance by transcriptionally regulating PTGS1 and ALOX5; loss of aryl hydrocarbon receptor (AhR) lowers prostaglandin E_2_ and leukotriene C_4_ and worsens airway inflammation (Maier et al., 2023). Training-immunity studies show that house-dust mite reprograms macrophages through the FPR2/TNF/2-HG/PGE_2_ EP2 axis, exaggerating secretion of tumor necrosis factor, CCL17, leukotrienes, prostaglandin E_2_ and interleukin-6, and establishing a CCL17-high M2-like phenotype that amplifies systemic allergy (Lechner et al., 2022). Cholesterol efflux is also relevant: in the OVA model, Tie2-hABCA1 mice overexpressing the transporter ABCA1 develop milder neutrophilic airway inflammation because cholesterol efflux in macrophages and endothelial cells lowers granulocyte colony-stimulating factor (G-CSF) production and restrains neutrophil recruitment (Dai et al., 2014). Multi-omics data reveal that ABCA1 is upregulated in macrophages during acute exacerbations but downregulated in chronic or severe asthma, suggesting a dual role that may involve extracellular-trap formation (Wang et al., 2025). Finally, coactin-like protein and gut-derived lipid metabolites such as 12,13-diHOME illustrate how perturbed lipid-handling pathways can skew macrophages toward either a prostaglandin D_2_–CRTH2-driven pro-inflammatory state or an IL-1β-high CD206-low phenotype that dampens IFN responses and increases the risk of childhood allergy and asthma (Pan et al., 2025; Lin et al., 2024).

#### 3.2.3 Subset-specific lipid metabolic signatures in M1 versus M2 macrophages

M1 macrophages rely on *de novo* fatty acid synthesis and accelerated glycolysis to secure rapid energy and anabolic precursors, whereas M2 macrophages depend on β-oxidation and oxidative phosphorylation for reparative and anti-inflammatory tasks (Batista-Gonzalez et al., 2019), the behavior of both subsets is finely tuned by cues within the lung micro-environment. Lipid pathways sculpt the phenotype and function of AMs and IMs alike (Batista-Gonzalez et al., 2019; Vanbever et al., 2019), thereby influencing both the escalation and the resolution phases of pulmonary inflammation (Kelly and O'Neill, 2015; Zhu et al., 2015). In the alveolar compartment, exogenous lipid species govern phagocytosis, maturation and secretory activity of AMs. High fat diet induced obesity enlarges the AM population after HDM challenge (Tashiro et al., 2017). In obese asthmatics, levels of surfactant proteins, especially surfactant protein A—decline; supplemental surfactant curtails eosinophil influx in murine airways, indicating that surfactant dysfunction contributes to obesity-related airway inflammation (Lugogo et al., 2018). AMs ingest and catabolize surfactant lipids, producing lipid-laden cells that accumulate in severe asthma and may exacerbate disease (Morales-Nebreda et al., 2015). Excess dietary lipids in obesity likely promote this buildup. During efferocytosis AMs remain anti-inflammatory, whereas uptake of necrotic debris releases pro-inflammatory danger signals (Hussell and Bell, 2014). Depletion of extracellular prostaglandin E_2_ heightens their phagocytic capacity (Pereira et al., 2018), and polyunsaturated fatty acids, such as eicosatetraenoic and docosahexaenoic acids—suppress tumor necrosis factor and interleukin-1β release, mitigating asthmatic inflammation (Mickleborough et al., 2009). Intracellular phospholipid turnover also shapes AM biology: macrophage-specific knock-down of phosphatidylinositol-3-phosphate 5-kinase lowers phospholipid stores, limits cellular expansion and aggravates lung inflammation (Kawasaki et al., 2017).

IMs, in contrast, display a less clearly defined lipid sensitivity. They are enriched in prostaglandin E_2_, prostacyclin, thromboxane A_2_ and hydroxyeicosatetraenoic acids, whereas AMs synthesize more leukotriene D_4_ and leukotriene E_4_, a metabolic divergence that may underlie their differential roles in asthma (Rouzer et al., 1982). Despite the presence of multiple lipid mediators that modulate macrophage driven inflammation, the detailed links among M1/M2 polarization, anatomical niche and lipid metabolic signatures, in both classical and obesity associated asthma, remain incompletely resolved. This complicates the design of metabolism targeted interventions.

### 3.3 Inflammation-resolving lipid mediators regulate macrophage polarization and their roles in asthma

Inflammation-resolving lipid mediators are thought to function as key signaling bridges between lipid metabolic reprogramming and macrophage polarization. AA and ω-3 polyunsaturated fatty acids are converted via lipoxygenase pathways into lipoxins, E-series and D-series resolvins, protectins, and maresins, a group of specialized pro-resolving lipid mediators that can limit further leukocyte recruitment, induce granulocyte apoptosis, enhance macrophage clearance of apoptotic cells, and drive a shift from proinflammatory to pro-resolving macrophage phenotypes (Barnig et al., 2018; Kytikova et al., 2019). Studies have shown that the biosynthesis of these endogenous inflammation-resolving lipid mediators and the signaling through their receptors are variably impaired in severe or treatment-refractory asthma. These abnormalities are associated with failure of airway inflammation to self-limit and with increased disease severity, providing a pathological basis for targeting resolution pathways in asthma (Barnig et al., 2018; Kytikova et al., 2019).

At the molecular level, lipoxins are among the most extensively studied inflammation-resolving lipid mediators. Lipoxin A_4_ (LXA_4_) is generated from AA through sequential 15- and 5-lipoxygenase activity, and its principal receptor is the G protein–coupled receptor ALX/FPR2 expressed on macrophages and other immune cells. In macrophage models, LXA_4_ markedly enhances nonphlogistic phagocytosis of apoptotic neutrophils and other targets via ALX/FPR2-mediated receptor internalization, and is regarded as a key signal that drives the resolution-phase macrophage phenotype (Maderna et al., 2010). In mouse RAW264.7 macrophages stimulated with LPS or IL-4, exogenous LXA_4_ suppresses activation of NF-κB p65 and the transcription factor IRF5 via the FPR2 pathway, downregulates M1 markers such as iNOS, IL-1β, and IL-6, and concomitantly upregulates M2-associated molecules including Arg1 and CD206, indicating that it can directly reprogram macrophages from a proinflammatory to an anti-inflammatory, pro-resolving state (Yuan et al., 2022). In a BALB/c mouse model of OVA-induced asthma, systemic administration of LXA_4_ significantly reduces eosinophil counts and Th2 cytokine levels in bronchoalveolar lavage fluid, attenuates airway smooth muscle hypertrophy and collagen deposition, and is accompanied by inhibition of STAT3 signaling in lung tissue, suggesting that LXA_4_ improves allergic airway inflammation *in vivo* by reshaping signaling pathways related to inflammation and remodeling (Liu et al., 2021). Lipoxin B_4_ (LXB_4_), another member of the lipoxin family, also exhibits pronounced pro-resolving activity in murine models of allergic rhinitis and asthma. Treatment with LXB_4_ reduces inflammatory cell infiltration and mucus production in both the upper and lower airways, decreases airway hyperresponsiveness, and accelerates the decline of inflammatory infiltrates and chemokine levels, overall favoring the resolution of allergic inflammation (Karra et al., 2015). The resolvin family likewise promotes resolution of asthma-related inflammation by modulating lung macrophage function. In an OVA-sensitized allergic airway inflammation model, administration of the DHA-derived mediator Resolvin D1 (RvD1) or its aspirin-triggered isomer at the peak of inflammation or during early resolution significantly reduces eosinophil and lymphocyte infiltration in the lung, attenuates airway hyperresponsiveness, and enhances the phagocytic capacity of lung macrophages toward IgG–OVA–opsonized particles, thereby shortening the duration of the resolution phase (Rogerio et al., 2012). In mice with LPS-induced acute lung injury, tail vein injection of RvD1 not only lowers pulmonary TNF-α, IL-1β, and IL-6 levels, but also increases M2 marker expression on F4/80^+^Ly6C^+^ recruited macrophages and enhances their phagocytic function. At the same time, RvD1 promotes apoptosis of these macrophages via an ALX-dependent FasL–FasR–caspase-3 pathway, reducing their accumulation in lung tissue and further accelerating inflammatory resolution (Xiang et al., 2021). The EPA-derived mediator Resolvin E1 (RvE1) exerts similar protective effects in an OVA mouse model that mimics acute exacerbations of chronic asthma. When administered after the acute challenge, RvE1 reduces inflammatory cell counts and cytokine levels in bronchoalveolar lavage fluid, markedly attenuates airway hyperresponsiveness and mucus secretion, and inhibits nuclear translocation of NF-κB p65 in lung macrophages, indicating that it terminates inflammatory amplification by suppressing proinflammatory transcriptional programs in macrophages (Flesher et al., 2014). Maresins are another class of inflammation-resolving lipid mediators synthesized by macrophages during the uptake of cellular debris. Among them, Maresin 1 (MaR1) shows robust protective effects in BALB/c mice with OVA-induced asthma. MaR1 treatment reduces inflammatory cell infiltration and goblet cell metaplasia in lung tissue in a dose-dependent manner, decreases IL-4, IL-5, and IL-13 levels in bronchoalveolar lavage fluid as well as serum OVA-specific IgE, and suppresses NF-κB activation and downstream cyclooxygenase-2 and intercellular adhesion molecule 1 expression in lung tissue, thereby alleviating allergic airway inflammation at multiple levels (Ou et al., 2021).

Taken together, findings from animal and cell-based studies indicate that inflammation-resolving lipid mediators such as lipoxins, resolvins, and maresins regulate both macrophage polarization and the course of asthmatic inflammation. On the one hand, they signal through ALX/FPR2 and related receptors to inhibit proinflammatory pathways including NF-κB and STAT, promoting polarization of recruited macrophages toward IL-10–high M2-like or resolution-phase phenotypes. On the other hand, they enhance nonphlogistic clearance of apoptotic granulocytes and allergen-opsonized particles and, in some models, induce programed cell death in a subset of macrophages during the resolution phase (Barnig et al., 2018). These actions reduce the overall inflammatory cell burden and release signals that support tissue repair. This continuous lipid signaling network is considered a critical regulatory node that determines whether asthmatic inflammation can be effectively terminated and whether an acute exacerbation can transition smoothly into a phase of structural and functional recovery (Barnig et al., 2018; Kytikova et al., 2019).

### 3.4 Sex differences in macrophage lipid metabolic reprogramming and their roles in asthma

Across the natural history of asthma, boys have a higher disease prevalence in childhood, whereas after puberty women predominate both in overall prevalence and in severe asthma phenotypes. This epidemiologic pattern suggests that sex hormones, and their regulation of innate immune cells, are likely to be important contributors to sex-based differences in asthma (Fuseini and Newcomb, 2017). In an OVA-sensitized and -challenged C57BL/6 mouse model, alveolar macrophages and bone marrow–derived macrophages from female mice express higher levels of M2-associated genes such as Arg1 after IL-4 stimulation, accompanied by upregulation of IL-4Rα and estrogen receptor ERα; oophorectomy attenuates this M2 polarization (Keselman et al., 2017). In human studies, *in vitro* polarization of peripheral blood mononuclear cells from patients with mild-to-moderate asthma shows that cells from female donors are more prone to acquire a classical M2 phenotype in response to IL-4 or IL-13, characterized by higher CD206 expression and increased levels of selected M2-associated chemokines, together with upregulation of IL-4Rα and relevant chemokine receptors (Becerra-Díaz et al., 2021). Furthermore, targeted metabolomic analysis in Chinese adults with asthma reveals marked sex differences in serum glycerophospholipid profiles, with multiple glycerophospholipids and lysophospholipids displaying sex-specific abundance patterns. These findings suggest that, under comparable disease conditions, lipid metabolic pathways are differentially reprogramed in men and women, which may in turn influence the availability of phospholipids and fatty acids as substrates for macrophages (Gai et al., 2018). In a model of nonallergic airway inflammation induced by inhaled multi-walled carbon nanotubes, alveolar macrophages from female mice are more likely to polarize toward an M2-like phenotype; inhibition of estrogen receptor signaling partially reverses this tendency. Lipidomic profiling in the same model shows sex-specific alterations in oxysterols and other cholesterol-derived metabolites in female macrophages, indicating that estrogens may participate in shaping macrophage polarization and function through the regulation of sterol metabolism (Ray et al., 2024).

### 3.5 Glycolysis–lipid metabolism coupling in macrophage polarization and asthmatic inflammation

During macrophage polarization, glycolysis and lipid metabolism are tightly coupled through the metabolic hubs citrate and acetyl-CoA, which together shape the inflammatory features of the M1 phenotype. In classically activated M1 macrophages, TLR and cytokine signaling markedly enhance aerobic glycolysis and the first half of the tricarboxylic acid (TCA) cycle, leading to mitochondrial accumulation of citrate. Citrate is then exported to the cytosol via the mitochondrial citrate carrier SLC25A1 and cleaved by ACLY into acetyl-CoA and oxaloacetate, thereby providing carbon skeletons and acetyl donors for *de novo* synthesis of fatty acids, cholesterol, and AA–derived lipid mediators (Infantino et al., 2014). In mouse bone marrow–derived macrophages and human monocytes stimulated with LPS or TNF-α, inhibition of SLC25A1 or ACLY significantly reduces fatty acid and prostaglandin synthesis, attenuates iNOS and COX2 expression, and diminishes the production of inflammatory mediators such as NO and PGE_2_, indicating that glycolysis-driven citrate export and ACLY activity constitute an upstream metabolic engine sustaining the lipid biosynthesis program of M1 macrophages (Infantino et al., 2014; Infantino et al., 2013). This glycolysis–citrate–ACLY axis also influences chromatin acetylation by providing acetyl-CoA for histone acetyltransferases, thereby helping to maintain an open chromatin state at M1-associated proinflammatory loci. In IL-4–polarized M2 macrophages, however, the same ACLY pathway, under the control of mechanistic target of rapamycin complex 1 (mTORC1) signaling, is repurposed to support histone acetylation at M2 marker gene loci (e.g., Mrc1 and Arg1), thus reinforcing an alternative activation program (Covarrubias et al., 2016). This mechanism has received partial support in asthma-related models: in OVA-induced allergic asthma mice and OVA-stimulated THP-1–derived macrophages, HIF-1α–driven enhancement of glycolysis, lactate accumulation, and protein lactylation are associated with increased pyroptosis of F4/80^+^ macrophages and aggravated airway pathology. Dexamethasone treatment suppresses the HIF-1α–glycolysis–lactate axis, markedly reducing glycolytic flux and inflammation in macrophages, suggesting that targeting core glycolytic pathways may indirectly limit downstream lipid synthesis and inflammatory mediator release (Chen et al., 2024). In models of inhaled HDM and other aeroallergens, macrophage-specific inhibition of glycolysis in alveolar macrophages shows that basal glycolytic activity helps restrain type 2 immune responses at steady state, but contributes to amplification of airway inflammation after allergen challenge, highlighting a dual regulatory role of alveolar macrophage glycolytic flux in both inflammatory amplification and immune homeostasis (Albers et al., 2025). Study in allergic asthma mice and MHS alveolar macrophages further demonstrate that the damage-associated molecular pattern S100A8/A9 enhances glycolysis and lactate production via TLR4/MyD88/NF-κB signaling, concomitant with increased ACLY phosphorylation and upregulation of M1 signature factors. Knockdown of S100A8 or S100A9 simultaneously suppresses glycolysis and proinflammatory polarization, suggesting that certain DAMPs can directly link TLR signaling to ACLY-dependent lipid synthesis to stabilize the M1 metabolic phenotype (Ji et al., 2024). In models of asthma exacerbation triggered by coexisting infection or pollutant exposure, formaldehyde exposure in OVA + LPS-induced mice and human peripheral blood–derived macrophages significantly upregulate LDHA expression and lactate levels, driving excessive secretion of TNF-α, IL-6, IL-1β, and NO. The glycolytic inhibitor 2-DG effectively reverses these effects, providing further evidence that high glycolytic flux continuously fuels lipid-driven inflammatory pathways in M1 macrophages, thereby exacerbating airway inflammation and lung dysfunction (Xuan et al., 2024). In a human segmental allergen challenge model of allergic asthma, single-cell RNA sequencing reveals that, compared with allergen-sensitized but asymptomatic controls, asthmatic patients exhibit stronger upregulation of glycolytic, matrix remodeling, and mucous metaplasia–related genes in lower airway epithelial and myeloid cells, accompanied by reprogramming of lipid mediator and growth factor networks. These findings indicate that glycolysis-driven carbon flux not only fuels local macrophage lipid synthesis but is also tightly coupled to airway structural remodeling (Alladina et al., 2023). In contrast, M2 macrophages do not simply switch off glycolysis; instead, they rebalance glycolysis with fatty acid oxidation (FAO) and mitochondrial oxidative phosphorylation (OXPHOS), channeling more acetyl-CoA into a complete TCA cycle and FAO-coupled energy metabolism (Covarrubias et al., 2016). This configuration maintains ATP production while preventing excessive glycolytic flux. In IL-4–induced M2-like macrophages, mTORC1 signaling promotes ACLY-dependent generation of nuclear acetyl-CoA to drive histone acetylation at M2 marker loci such as Mrc1 and Arg1, while simultaneously sustaining relatively high mitochondrial respiration and FAO activity. Under conditions of high ATP, elevated citrate, and sufficient NADH, classical product feedback inhibition of rate-limiting glycolytic enzymes such as phosphofructokinase constrains further increases in glycolysis (Viola et al., 2019; Covarrubias et al., 2016).

Within allergic airway inflammation, this shift toward FAO and OXPHOS interweaves with remodeling of the lipid mediator landscape. In macrophages, the AhR transcriptionally regulates key enzymes such as PTGS1/COX-1 and ALOX5/5-LOX, thereby fine-tuning the balance between PGE_2_ and LTC_4_ production (Maier et al., 2023). Loss of AhR function leads to concurrent downregulation of both classes of lipid mediators and is associated with exacerbated airway inflammation, suggesting that a moderate level of prostaglandin and leukotriene production is itself an integral component of the anti-inflammatory and tissue-protective functions of M2-like macrophages (Maier et al., 2023). In an OVA-induced neutrophilic airway inflammation model, Tie2-hABCA1 transgenic mice, which exhibit enhanced cholesterol efflux in macrophages and vascular endothelial cells, show markedly reduced G-CSF production and neutrophil recruitment. This indicates that lipid efflux and membrane cholesterol content can reprogram macrophage inflammatory sensitivity, providing a metabolic entry point for limiting excessive lipid accumulation and inflammatory amplification in an M2-skewed context (Dai et al., 2014). In addition, the TCA-derived metabolite itaconate not only modulates the M1 metabolic checkpoint by inhibiting succinate dehydrogenase, but also exerts immunoregulatory effects in dust mite–induced allergic airway inflammation (Li et al., 2024). Augmenting endogenous itaconate or administering cell-permeable itaconate derivatives alleviates type 2 inflammation and suppresses T cell responses, indirectly supporting the concept that strengthening oxidative metabolism and its downstream metabolites in M2 or regulatory macrophage contexts may control asthma inflammation by dampening excessive glycolysis and lipid-driven inflammatory pathways (Li et al., 2024). Collectively, these findings suggest that glycolytic flux, via citrate and ACLY, directly fuels the lipid biosynthesis and lipid mediator production required for M1 polarization, whereas in M2 macrophages, FAO- and OXPHOS-dominant metabolic programs provide abundant energy and feedback inhibition that restrain glycolysis, favoring anti-inflammatory and reparative phenotypes (Infantino et al., 2014). In future strategies aimed at metabolically reprogramming macrophages in asthma, a key challenge will be to precisely modulate this coupled glucose–lipid metabolic axis according to disease etiology and stage—for example, downregulating M1-associated SLC25A1/ACLY activity during acute exacerbations or infection-related phenotypes, while optimizing ABCA1-mediated lipid efflux and M2 oxidative metabolism during chronic remodeling—to enable truly phenotype-guided therapy (Albers et al., 2025; Infantino et al., 2014).

## 4 Therapeutic manipulation of macrophage immunometabolism in asthma

### 4.1 Pharmacologic suppression of glycolytic flux: mechanistic basis and pre-clinical efficacy

Recent work has identified excessive reliance on glycolysis by pulmonary macrophages as a metabolic driver of airway inflammation (Table 2). Blocking this pathway significantly alleviates asthmatic pathology. The hexokinase inhibitor 2-DG is the most widely used glycolytic antagonist. In OVA-sensitized mice, 2 DG suppresses TLR2 and HIF-1α driven glycolysis in alveolar macrophages, thereby preventing pyroptosis, oxidative-stress signaling and airway hyper-responsiveness, and ultimately reducing allergic inflammation (Sha et al., 2023). 2 DG also reverses the trained-immunity phenotype of bone-marrow macrophages after enterovirus A71 infection, leading to a marked attenuation of Th2 and Th17 responses in a HDM model (Chen et al., 2021). In asthma complicated by respiratory infection, exposure to fatty acids increases LDHA expression and lactate production in macrophages, which in turn drives excessive secretion of TNF-α, IL-6, IL-1β and NO; 2 DG abolishes these pro-inflammatory effects in both animal models and patient-derived macrophages (Xuan et al., 2024). To enhance selectivity, Albers and colleagues used two macrophage-targeted approaches: conditional deletion of the glycolytic program with LysM-Cre mice and intratracheal delivery of 2 DG-loaded liposomes. Both strategies lowered type-two cytokine infiltration and bronchoconstriction in several models, confirming the central role of alveolar macrophage glycolysis in asthma (Albers et al., 2025). Metabolic reprogramming can also be achieved without direct enzyme inhibition. Molecular hydrogen, administered as hydrogen-rich saline, restores the balance between glycolysis and OXPHOS in patient monocytes and in OVA mice, downregulates hexokinase (HK), phosphofructokinase (PFK) and HIF-1α, rescues mitochondrial complex I and III activity and normalises ATP production, which together diminish eosinophil infiltration and airway resistance (Niu et al., 2020). These findings establish overactive glycolysis in macrophages as a metabolic engine of airway inflammation and demonstrate that pharmacological or genetic interruption of this pathway affords reproducible protection in pre-clinical asthma. They further indicate the need to discover small-molecule inhibitors that are more selective and drug-like than 2 DG.

**TABLE 2:** Applications of strategies targeting macrophage metabolic pathways in asthma therapy.

Intervention	Molecular target	Experimental model	Pharmacological effects	References
2-DG	Glycolysis	OVA-sensitized mice	Suppressed glycolysis, pyroptosis and oxidative stress; lowered airway hyper-responsiveness and allergic inflammation	Sha et al. (2023)
	Glycolysis	BMDMs from recovered EV-A71-infected mice	Diminished Th2/Th17 cytokines and airway resistance	Chen et al. (2021)
	Glycolysis	Formaldehyde exposed OVA model; Human monocyte-derived macrophages	Reversed lactate accumulation and excessive TNF-α, IL-6, IL-1β, NO release	Xuan et al. (2024)
Macrophage-targeted 2-DG liposomes	Glycolysis	Aero-allergen challenge mice	Lowered type 2 cytokines and bronchoconstriction	Albers et al. (2025)
Hydrogen rich saline	Energy metabolism regulation pathways	OVA asthma mice model and PBMCs from patients	Restored mitochondrial complex I/III activity, boosted ATP, reduced eosinophilic inflammation	Niu et al. (2020)
Etomoxir; Ranolazine	CPT1	OVA and HDM mouse asthma model	Blocked FAO, diminished inflammatory-cell influx and airway hyper-responsiveness	Al-Khami et al. (2017)
Rosiglitazone	PPARγ	Chronic OVA model	Suppressed eosinophilic inflammation and airway remodeling via TLR4-NF-κB inhibition	Lee et al. (2016)
Pioglitazone	PPARγ	Cockroach allergen asthma mice model	Reduced AhR and cytokines, efficacy comparable to dexamethasone	Narala et al. (2007)
Fenofibrate	EHHADH/peroxisomal β-oxidation pathway	LPS-primed Neutrophilic asthma mice model and BMDMs	Activated peroxisomal FAO, restrained M1 polarization and neutrophil recruitment	Chen et al. (2025)
FABP5 deficiency	FABP5	Fabp5^−/−^ mice	Loss of FABP5 caused long-chain unsaturated FA build-up, enhanced M2 polarization and worsened airway inflammation	Hou et al. (2022)

### 4.2 Targeting fatty-acid and cholesterol circuitries to restore pulmonary lipid homeostasis

Imbalance in macrophage lipid handling is increasingly recognized as a contributor to asthma onset and chronic inflammation. Several animal studies show that direct intervention in fatty acid oxidation or in lipid-sensing pathways markedly improves airway inflammation and remodeling. In OVA and HDM models, activity of carnitine-palmitoyl transferase 1 (CPT1), a rate-limiting enzyme in β-oxidation, rises along with inflammation. Pharmacological inhibition of CPT1 with etomoxir or ranolazine swiftly reduces fatty acid flux, inflammatory-cell infiltration and airway hyper-responsiveness (Al-Khami et al., 2017). Intranasal rosiglitazone, which activates macrophage PPAR-gamma, weakens persistent eosinophilic inflammation and suppresses TLR4 and NF-κB driven airway remodelling (Lee et al., 2016). Similarly, the thiazolidinedione pioglitazone shows efficacy comparable to dexamethasone in a cockroach-allergen model, lowering airway hyper-responsiveness and multiple pro-inflammatory cytokines (Narala et al., 2007).

Neutrophilic asthma presents a different metabolic signature. Downregulation of peroxisomal β-oxidation enzymes, such as EHHADH, disturbs macrophage lipid metabolism, promotes M1 polarization and recruits neutrophils through TNF-α and IL-6, thereby intensifying airway inflammation. Activation of the peroxisomal pathway with the agonist fenofibrate suppresses M1 polarization and may relieve neutrophilic inflammation (Chen et al., 2025). Not all interventions are beneficial, however. Deletion of the lipid-binding protein FABP5 aggravates asthma rather than relieving it. Accumulation of long-chain unsaturated fatty acids skews Fabp5-deficient macrophages toward an M2 phenotype, exacerbating airway inflammation in the OVA model (Hou et al., 2022). These observations underline the need to balance inhibition of fatty acid oxidation with preservation of essential lipid homeostasis when designing therapies that target macrophage lipid metabolism.

Taken together, macrophage immunometabolism-based interventions have shown promising potential to suppress airway inflammation and remodeling in animal models, including glycolysis inhibitors, fatty acid oxidation modulators, and small molecules targeting cholesterol efflux or nuclear receptor signaling. However, translating these preclinical findings into patient care faces several critical challenges. Most currently available metabolic inhibitors act systemically and cannot be easily restricted to alveolar or monocyte-derived macrophages without simultaneously perturbing airway epithelial cells and lymphocytes; moreover, prolonged or excessive pathway blockade may impair pathogen clearance and trigger compensatory metabolic circuits. These factors collectively limit the feasibility of directly repurposing existing metabolic drugs for asthma treatment (Qin et al., 2024). In parallel, asthma in the clinic encompasses multiple endotypes, including type 2 (T2)-high versus T2-low, eosinophil-versus neutrophil-predominant, and obesity-associated phenotypes. These endotypes are characterized by heterogeneous inflammatory drivers and variable responses to current biologics, which makes it unlikely that a single metabolic target will be effective across all patient groups (Sim et al., 2024; Villaseñor et al., 2021). Thus, the key to future metabolism-targeted therapies lies in integrating clinical endotypes with immunometabolic signatures. High-throughput metabolomics and other omics platforms will be essential to establish reproducible, quantifiable biomarkers that can identify patient subgroups most likely to benefit from inhibiting M1 glycolysis or reprogramming M2 lipid metabolism, and to enable dynamic monitoring of on-target drug effects (Turi et al., 2018; Papamichael et al., 2021). Studies have already identified glycolytic intermediates, lipid species, and energy metabolites in plasma, urine, and various airway-derived samples from adult and pediatric asthma patients that associate with disease onset, endotype, and severity, providing a methodological foundation for glucose–lipid metabolism-based stratified therapy (Villaseñor et al., 2021; Papamichael et al., 2021). Synthesizing these multi-omics and immunometabolic data, current evidence supports such strategies primarily at the conceptual level. In future clinical trials, patients could be prospectively stratified according to glycolytic and lipid metabolic biomarkers into distinct therapeutic arms, with one arm focusing on inhibition of M1-related glycolysis and the other on modulation of M2-associated lipid metabolism. These regimens could be combined with local and/or cell-targeted drug delivery approaches, which may allow more precise macrophage metabolic intervention in asthma while minimizing systemic metabolic adverse effects.

## 5 Conclusion and future perspectives

This review has systematically examined the lineage composition, polarization plasticity, metabolic reprogramming, and therapeutic relevance of pulmonary macrophages in asthma. Embryo-derived TRAMs, inflammation-recruited MoAMs, and IMs act in concert, filling complementary roles at different disease stages. Under pro-inflammatory cues such as IFN-γ and LPS, they shift toward an M1 phenotype that is marked by high expression of iNOS, IL-6, IL-12, and TNF-α. In contrast, IL-4, IL-13, or IL-10 drive them toward an M2 phenotype characterized by upregulation of MRC1, CD163, and Arg-1 and by amplification of type 2 responses through CCL17 and CCL22. Phenotypic transitions occur together with a re-arrangement of metabolic circuits. Allergen exposure rapidly engages aerobic glycolysis, and a lactate–HIF-1α feedback loop amplifies inflammation. The SREBP–ACLY–FAS axis strengthens fatty acid and cholesterol synthesis, while cyclo-oxygenase and 5-lipoxygenase balance prostaglandin and leukotriene production. Fatty acid oxidation and OXPHOS provide sustained energy for M2 cells. The extent and persistence of inflammation are further shaped by ABCA1-mediated cholesterol efflux and by remote lipid signals from the gut microbiota. Interventions that target these metabolic features have shown efficacy in animal models. Glycolysis blockade with 2-DG, either systemically or via macrophage-directed liposomal delivery, markedly reduces airway hyper-responsiveness and inflammatory-cell infiltration. Inhibition of CPT1 or activation of PPAR-γ and peroxisomal β-oxidation redirects lipid flux, restores the M1/M2 balance, and limits airway remodeling. Restoring the function of transporters such as ABCA1 and FABP5 helps to re-establish pulmonary lipid homeostasis.

Integrating lineage, polarization, and metabolism underscores the pivotal position of macrophages in asthma pathogenesis and suggests that precise control of glycolytic and lipid pathways may offer new options for refractory disease. Despite considerable progress, single-cell and spatial omics have revealed continuous transitional states rather than strict separation between tissue-resident and monocyte-derived macrophages at sites of inflammation. The traditional M1/M2 dichotomy no longer explains their functional diversity within the same lesion. At the same time, single cell RNA sequencing and high dimensional cytometry reveal multiple macrophage clusters within the broad M1 and M2 categories. Integrating these datasets with metabolic flux tracing and metabolomics in human samples will be essential to define metabolically distinct macrophage states that actually drive steroid resistance, airway remodeling or resolution. Such multidimensional maps are likely to provide the framework for precision interventions, in which patients are stratified by macrophage metabolic signatures and then allocated to glycolysis focused, lipid remodeling focused or combined therapies rather than receiving a one size fits all approach. High-resolution lineage tracing combined with metabolic-flux analysis will be required to clarify how inflammation resolves into repair. Most knowledge of macrophage polarization still derives from murine models, yet humans and mice differ substantially in gene expression and metabolic wiring. Direct translation is therefore challenging, especially when metabolic intervention is used to reprogram macrophages. A pressing need exists to characterize metabolic imbalances in immune cells from patients with asthma, both to discover new biomarkers for disease stratification and to pinpoint therapeutic targets.
